# Identifying Topics in Microblogs Using Wikipedia

**DOI:** 10.1371/journal.pone.0151885

**Published:** 2016-03-18

**Authors:** Ahmet Yıldırım, Suzan Üsküdarlı, Arzucan Özgür

**Affiliations:** Department of Computer Engineering, Boğaziçi University, İstanbul, Turkey; Katholieke Universiteit Leuven, BELGIUM

## Abstract

Twitter is an extremely high volume platform for user generated contributions regarding any topic. The wealth of content created at real-time in massive quantities calls for automated approaches to identify the topics of the contributions. Such topics can be utilized in numerous ways, such as public opinion mining, marketing, entertainment, and disaster management. Towards this end, approaches to relate single or partial posts to knowledge base items have been proposed. However, in microblogging systems like Twitter, topics emerge from the culmination of a large number of contributions. Therefore, identifying topics based on collections of posts, where individual posts contribute to some aspect of the greater topic is necessary. Models, such as Latent Dirichlet Allocation (LDA), propose algorithms for relating collections of posts to sets of keywords that represent underlying topics. In these approaches, figuring out what the specific topic(s) the keyword sets represent remains as a separate task. Another issue in topic detection is the scope, which is often limited to specific domain, such as health. This work proposes an approach for identifying domain-independent specific topics related to sets of posts. In this approach, individual posts are processed and then aggregated to identify key tokens, which are then mapped to specific topics. Wikipedia article titles are selected to represent topics, since they are up to date, user-generated, sophisticated articles that span topics of human interest. This paper describes the proposed approach, a prototype implementation, and a case study based on data gathered during the heavily contributed periods corresponding to the four US election debates in 2012. The manually evaluated results (0.96 precision) and other observations from the study are discussed in detail.

## Introduction

Twitter [1] is the most popular microblogging system in the world with over 280 million active users tweeting around 40K posts/s [2]. It serves as a collective platform where users tweet (post) anything about anything [3], such as current events, sports, politics, health, conferences, personal life, etc. Most tweets are publicly visible. Users can view the tweets of others by *follow*ing users. The tweets of followed users are published in a feed that is compiled on the *follow*ers home page. Users can also search for tweets using keywords, which is often done during a major event such as a riot or natural disaster. However, for any given topic, the large volume of tweets, the high number of users, and the characteristics of the short and terse posts makes it difficult to grasp the larger picture of the event or situation.

Most tweets are posted in an uncoordinated manner: users post similar or different aspects of the same issue with no focus or organization provided by the platform. Once a tweet is posted, an observer of that post can take further action, for example, propagating it by *retweet*ing (reposting) the tweet, which makes the tweet visible to the retweeter’s followers. Retweets can be further retweeted. This action has the potential of greatly increasing the visibility of particular tweets, especially when popular users (those with many followers) are involved. When a significant event occurs it is often marked by a spike in tweets on the same or similar subjects.

Twitter is a powerful resource for gaining insight in to what concerns its users. Collections of tweets can reflect information about the public, such as their opinions, perceptions, emotions, and much more. This information can be utilized in numerous ways, such as news generation, policy making, campaign management, marketing, and disaster management.

This work focuses on identifying the topics of a collection of tweets. The data source, is a set of tweets, where each tweet may be related to one or more topics, or to no particular topic at all. Furthermore, aspects of a topic are likely distributed over different tweets, thus an aggregate approach is considered. The high volume of tweets, the limited size of posts (140 characters), and posting characteristics (Section *About microblog posts*) require special processing methods.

This work, proposes an approach to topic identification for a collection of tweets. It takes a set of tweets and generates a ranked list of topics. Topics are identified with English language Wikipedia articles. A topic corresponds to a user readable Wikipedia article title (i.e. Christianity and Abortion), rather than a set of keywords that is common in approaches (Section *Related work*). Wikipedia was chosen as a topic resource since it both spans across all human interest and is up-to-date due to constant contributions of new material. This up-to-date aspect is crucial since Twitter posts are about current events, issues, and people. Unlike conventional encyclopedias, Wikipedia contains more topical articles like (“Death of Freddie Gray” or “Emailgate”). Being up to date is very significant, since Twitter tends to be dominated with content that is temporarily relevant, often fading in importance in a single day.

This paper presents an approach to topic identification, a prototype implementation, and the results of this approach using data gathered during the 2012 US Election debates (when Twitter was very heavily used [4–7]). The televised debates were moderated, constraining both the time and and varieties of topics available to the speakers. On the other hand, those who were following the debate, were freely tweeting on all subjects. Since the campaign teams focus on conveying specific messages on particular topics, knowing which issues resonated with the audience should be an obvious point of inquiry. Another matter of interest is determining which unexpected discussions emerge from a particular subject.

The topics identified for the four debates were evaluated by human evaluators with a precision of 0.96 and inter annotator agreement of *F*_1_ measure *F*_1_ = 1.

The main contributions of this work are:

- An approach to identify human-readable and domain-independent topics for sets of tweets.
- Resources
  - Manually annotated sets of tweets with corresponding topics (30 sets of 6000 tweets each)
  - Inverse document frequency (*idf*) scores of a public stream over 5 days, which is useful in assessing the values of tokens in Twitter.
  - Term frequency (*tf*) and *idf* scores of words in a Wikipedia snapshot.

The remainder of the paper is organized as follows: The following two sections provide a background to and an overview of related works. Then, the next two sections present the proposed model and its implementation, followed by two sections presenting the results using data gathered during the 2012 US presidential debates, and a discussion of this approach and the results. The final section presents future work and conclusions drawn from this study.

## Background

This section provides foundational information key to understanding the work. We refer to a tweet as microblog post since in theory, the approach can be applied to other short message systems as well.

### About microblog posts

Microblog users tend to write short, expressive, and distinctive messages. Especially if users want to be noticed, they must make effective use of the limited space provided. For instance, users use hashtags intending to make their posts share the same context with posts using the same hashtag (hashtags start with # sign). Consider the tweet “Obama: Take some of the money we’re saving as we wind down two wars to rebuild America. #debate”. This tweet declares that its subject is part of the context of the debate. It quotes Obama’s sentence. This way, the author makes a connection between Obama’s words and the context of the debate. Further, he can add his opinion on the subject if he wants.

Users intend to post frequently about various topics and this makes microblogs a useful source to investigate the intentions of a particular group.

Regardless of potential usefulness, it may be difficult to process tweets because of different posting styles. Tweets are full of abbreviations (hny for honey, omg for oh my god), misspellings, jargon, profanity, and twitter specific syntax (i.e. hashtags like #cantwait and user references @camanpour). They are often one or more fragments of sentences. These factors make it difficult to process posts with conventional NLP approaches [8]. Furthermore, since, rather than well formed contributions from a collaborative effort, contributions are from a wide range of mostly unrelated users, identifing topics of these contributions by processing a collection of such posts by partitioning and then aggregating would be a better process than conventional NLP approches.

### Wikipedia

Wikipedia includes over four million articles about many topics. Articles are formed by human contribution and collaboration. Wikipedia policy dictates that the title of an article is either a name or a description of the subject of the article [9]. Therefore, the title of a Wikipedia article can be used to refer to similar content. We propose to use the titles of Wikipedia articles to represent topics.

### Vector space model and document similarity

The *tf-idf* vector space model represents a document -in a set of documents- as a vector [10]. Each element in the vector has a value of a word that gives the strength of the relation between the word and the represented document.

The strength of a word in a document is obtained by *tf* *idf. *tf* (term frequency), is the number of times the word occurs in that document and *idf* (inverse document frequency) is obtained as $idf=log(\frac{number\;of\;all\;documents}{number\;of\;documents\;which\;include\;the\;word})$. The similarity between two documents is the cosine of the angle between the two representative vectors.

### WordNet Lexical Database

WordNet [11] is a lexical database of English words and phrases, which is widely used in artificial intelligence, machine translation, text classification, text summarization, and text analysis tasks. WordNet has over 115 thousand groups of words called synonym sets (syn-sets). A synonym of a word or phrase is another word or phrase that has the same meaning. Each syn-set in WordNet has a different and unique meaning. For example, the words “car”, “auto”, “automobile”, “machine”, and “motorcar” form one of the syn-sets in WordNet. In our work, we used WordNet to identify the syn-sets of words that occur in microblog posts. This helps to identify Wikipedia pages that contain synonyms of the words that occur in the microblog posts.

## Related work

The approaches for determining the topic of microblogs differ in terms of whether they focus on a single or multiple posts, the use of external resources (such as WordNet), the methodology, and how they consider the resulting topics.

Some of the work focuses on determining the topic of a single microblog post. Given the nature of how people post, this is quite a challenge. Alternatively, some approaches attempt to determine the topic of a set of posts, such as within a given time interval or geographical region. We refer to these approaches as single post processing and collective processing approaches respectively.

Some approaches rely on external resources or meta information to assist in determining topics, whereas others do not. Typical external resources provide information about the syn-sets of words (e.g. WordNet), encyclopedic information (e.g. Wikipedia), slang (e.g. Twitter Dictionary Guide [12]). Meta information is information such as the language, creation-time, location, etc. of the post, which can also be useful in determining topics. For instance, the frequency change of words close together in time may indicate an emerging topic.

In terms of identified topics, typically sets of keywords or phrases, or, one or more representative microblog posts are provided. The common methods are: LDA (Latent Drichlet Allocation), classification of posts according to several features, clustering a graph where posts are nodes and edges are weighted according to a similarity measure between the two posts. Similarity measures and classification features are based on the number of common words, and the semantic relatedness of words or phrases -computed by using external data such as WordNet or Wikipedia- among posts. Meta information about a microblog post are also used in such measures and features. Another research track uses manually defined, or automatically obtained word or phrase sets for identification. A set represents a topic of interest. The number of times that particular words or phrases in that set appear in the microblog posts measures the strength of the topic. Lastly, another type of research uses common consecutive words to build a topic identifier using a phrase.

We will refer to related works in terms of these general criteria. We remind the reader that in terms of these criteria our approach provides a human readable ranked list of topics corresponding to Wikipedia article titles for a given set of microblog posts. It utilizes Wikipedia articles as an external resource. The approach essentially determines the similarity of a set of microblog posts to a set of Wikipedia articles. This approach is a collective processing approach.

For identifying topics, the most widely utilized collective processing methods in the literature are probabilistic topic models. Among these approaches, LDA based approaches have been proposed by several works [13–17].

In probabilistic topic modeling, documents are assumed to be a multinomial mixture of hidden topics, while the topics are represented as a probability distribution over a number of words. LDA separates related words into sets, which are the considered as topics. Identifying the underlying concept associated with sets of words usually requires additional -possibly manual- analysis. For instance, [17] gives the set “{rob, moon, love, twilight, gaga, lady, #nowplaying, adam, lambert, fans, kris, chirs, brown, song, beyonce, download, live, mixtape, music}” as a topic. But, this set does not fully explain what the topic is. To make it refer to a concept, the authors further process and automatically assign it to “arts” category from a set of pre-defined categories.

A number of collective processing approaches have been proposed for identifying microblog topics by considering the highly temporal nature of the posts. [18–24] base topic identification on the frequency of change in words and hashtags. Regarding the generation of possible topics, these approaches either measure the frequency change of words or create a representative set of posts related to these words.

Other collective processing approaches proposed by [25–27] also provide sets of microblog posts as possible topics. However, they utilize similarity measures among microblog posts to identify topics. Either applying latent semantic analysis (LSA) based vector space models to measure the similarity between two posts, or measuring the similarity among words and phrases through other metrics. An example of such a metric is the distance between two Wikipedia pages on a link graph. The pages are identified by the content of the posts. With these approaches, identifying the underlying concept still requires further processing.

[28] proposed a collective processing approach which uses WordNet [11] for identifying potential topics. The authors seek matches between the words in posts and the words in WordNet syn-sets while manually investigating results, discovering that temporal occurrences of patterns of hashtags over time may indicate different WordNet classes.

Another collective processing approach is the study by Lansdall-Welfare *et al*. [29]. They manually defined keywords for four different classes of moods. Counting how many of these keywords appear in the post set determined the strength of the moods. They show that, they were able to identify Christmas, Halloween, Valentines Day, and Easter, along with attributed moods for these days. They were also able identify negative mood such as what occurred after cuts to public spending. In the domain of health, [30] manually specified the words and regular expressions. Each group of words and regular expressions indicate a sickness. Importance of a sickness to the public can be measured by counting the matching words and regular expression in a post set.

In contrast to these approaches, [31] was able to automatically obtain indicative words in the domain of health. The authors extracted words (e.g. symptoms) from health related Wikipedia articles to identify collective public health trends. Counting the words related to a particular sickness in a post set measured the importance of that sickness to the public. They applied the approach on health related post sets and showed that they could identify sicknesses like flu and ice-cream headaches. These approaches show that, using external data sources for collective topic identification in posts is possible and promising.

Sharifi *et al*. [32] proposed a collective processing approach that was built on a summarizing phrase, a given post set, and a phrase of interest. The algorithm builds the summarizing phrase towards the left and right considering the phrase of interest placed at the center, building phrases using common consecutive words. But, since the phrase is formed from microblog posts, sometimes it may not be grammatically correct or even meaningful. We believe that using descriptive topic sources like Wikipedia for describing the post set gives more satisfying and possibly precise results. Our proposed approach differs from this approach since ours benefits from the way Wikipedia articles are titled. Our approach seeks the most relevant Wikipedia article and returns its title because we believe, based on the site’s own guidelines, that an article’s content is represented by the title of the article.

Several approaches try to semantically enhance short texts or microblog posts to better identify the content of microblog posts. [33–37] link the text of single microblog posts or parts of single microblog posts to external resources. [34, 35] considers Wikipedia titles and link structures, while [36] considers other data sources such as MusicBrainz, City DB, Yahoo! Stocks, Chrome, and Adam in addition to Wikipedia titles. [37] considers Wikipedia article bodies, links, and anchors in addition to titles for topic identification in a single-microblog post. Considering the limited length of microblog posts which leads to a limited context, and discarding the descriptive content of Wikipedia article bodies may lead to less inclusive and less descriptive topics as we show in *Comparison of processing single-microblog posts and microblog post sets* section while also examining some cases by comparing the results between an approach that aggregates what [34, 35] returns and our own proposed approach.

To summarize, our proposed approach both accounts for the collective processing of microblog posts and external topic source usage. Approaches that use external sources such as Wikipedia try to find representative elements for only one microblog post or a piece of the microblog post. Our approach differs from these approaches in that it processes the whole set considering the global context in the set itself. To the best of our knowledge, no approach exists that collectively processes and identifies topics in a microblog post set using external sources such as Wikipedia article contents and addressing Wikipedia titles as topics.

## Approach

This work proposes, BOUN-TI, an approach to identify topics of sets of microblog posts. Here, topics are in the sense of the underlying concepts of a given set of microblog posts. The set of topics are drawn from the titles of Wikipedia articles, as they encompass most topics of human interest, Wikipedia itself is comprised entirely of user generated content and includes a broad spectrum of articles including very current ones [36]. The issues needed to be resolved related to how to select relevant articles for a given set of posts. The idea is to locate the articles that are closest to the content being produced in the posts.

Content comparison between a Wikipedia article and a collection of tweets helps illustrate the highly distributed nature of microblogs. For instance, if a post set that consists of many posts including the word “abortion”, many others including “Christianity”, and fewer including both is given, and if this set is processed collectively, the Wikipedia topic “Christianity and Abortion” would be a result instead of the separate topics “Abortion” and “Christianity” which is the case in single-microblog post or short document processing approaches that use external sources [33–37]. These approaches aim to identify parts of short texts and link them to sources like Wikipedia in order to enrich their limited content semantically. But, they miss the overall post set level context due to the short lengths of individual posts. A single post is unlikely to contain sufficient information to indicate the topic(s) that users are contributing on. Rather, it may provide a valuable part of the larger context. Information about the topics of interest will be spread over a large number of posts. By examining groups of posts as a whole we expect to identify terms relevant to the topic. Furthermore, we expect that the significant terms will also appear in Wikipedia articles—the encyclopedia by the people, for the people.

We define task of topic identification as a task of information retrieval: “given a microblog post set, return the best representing topics in a set of topics”. The input microblog post set may be retrieved by a set of words from the microblog application programming interfaces (APIs).

**Fig 1:**
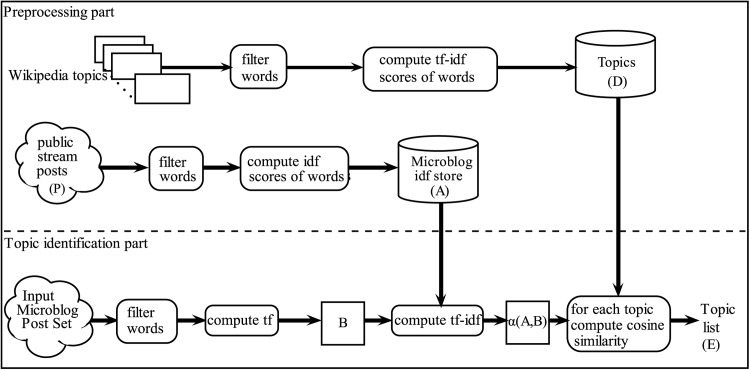
Overview of the topic identification approach. Sets correspond to sets defined in the formal explanation. Preprocessing part: Preparation of resources for topic identification. Topic identification part: Topic identification for a specific set of microblog posts. The approach in this part uses resources created in the preprocessing part.

Fig 1 shows an overview of the proposed approach. The approach has two parts: the preprocessing part and topic identification part. The preprocessing part computes and stores values needed for similarity computation. The topic identification part compiles the computation related to the input set.

Prior to computing, in both parts, some filtering is required to remove content that is not useful for the computation. For example, profanity, special syntax, abbreviations, etc. are not relevant to identifying topics in our context since Wikipedia articles are written more clearly. These operations are represented as the *filter words* boxes in the Fig 1.

The basic idea is to apply the *tf-idf* vector space model to determine topics associated with a set of microblog posts. In the preprocessing part, *tf-idf* values of all Wikipedia words are computed. The body of an article is considered to be a complete document in this computation. The idf values of words in Wikipedia pages are computed as given in “Vector space model and document similarity” section. The values are saved and made accessible as the “Topics” list as given in Fig 1.

Other computations made in the preprocessing part are the *idf* values of words in a microblog post set. This computation is not insignificant, since tweets are an ever increasing set of posts and we assume that the input post set would be insufficient since it may not cover sufficient words used in tweets in order to derive how common a word is. The nature of the query may also result in a specific set of words or the time interval may limit coverage. Therefore, we decided to consider a set that is not based on a query and one that has a relatively long time interval. For this, we collected data from the general public stream over a longer time span, five days.

The *idf* value of a word *w* in the the longer public stream set *P* is:

$$\begin{array}{c} \text{idf}\left(w,P\right)=log(\frac{\left|P\right|}{max(|\{d\in P:w\in d\}|,1)}) \end{array}$$ (1)

Each element (document) in P is a set of words that correspond to a microblog post. Since, the tweet sets that we are inspecting are not collected during the same time period as the Public Stream set (P) was collected, it is possible to encounter tokens in the tweet sets that do not exist in P. Such tokens are considered to be rare in the microblog environment. Since rare tokens are assumed to be more informative according to idf, we assign their document frequency to one in P, so that they get high values in the idf computation. The *idf* values of words are stored as “Microblog idf store” as shown in Fig 1.

In the topic identification part, similarities between an input set and each of the articles are computed. For computing *tf-idf* values of words of the input set, a modified *tf-idf* vector space model is applied. All posts are considered to be one document for computing the *tf* values of words. The *idf* values of words are taken from the “Microblog idf store”.

A formal explanation of the topic identification part is given below.

Let N be the set of natural numbers, $R_{\geq 0}$ be the non-negative real numbers. A string is a sequence of alphanumeric characters. Let *T* be a set of all strings.

Let set *A* represent the set of tokens (strings) and their corresponding *idf* value pairs which is the “Microblog idf store” in Fig 1. *A* is a set of pairs (*t*, *r*) where *t* ∈ *T*, $r\in R_{\geq 0}$, $A\subset T\times R_{\geq 0}$, and *A* meets the constraint ¬∃(*t*, *r*_1_), (*t*, *r*_2_) [*r*_1_ ≠ *r*_2_∧(*t*, *r*_1_)∈*A*∧(*t*, *r*_2_)∈*A*].

Let set *B* represent the set of tokens (strings) and their corresponding frequencies (*tf* values) in the input microblog post set which is the “Input microblog post set” in Fig 1. *B* is a set of pairs (*t*, *n*) where *t* ∈ *T*, n∈N, B⊂T×N and *B* meets the constraint ¬∃(*t*, *n*_1_), (*t*, *n*_2_) [*n*_1_ ≠ *n*_2_∧(*t*, *n*_1_)∈*B*∧(*t*, *n*_2_)∈*B*].

Let set *C* represent all possible tokens and their possible corresponding *tf-idf* values. Let set *D* represents the set of all Wikipedia articles which is the “Topics” store in Fig 1. *C* is a set of pairs (*t*, *r*) where *t* ∈ *T*, $r\in R_{\geq 0}$, $C\subset T\times R_{\geq 0}$. *D* is power set of *C*. D=P(C). And *D* meets the constraint ∀*d* ∈ *D*¬∃(*t*, *r*_1_), (*t*, *r*_2_) [*r*_1_ ≠ *r*_2_∧(*t*, *r*_1_)∈*d*∧(*t*, *r*_2_)∈*d*].

Given sets *A*, *B*, and *D*, set *E* is computed. Set *E* represents Wikipedia articles and their corresponding similarity values to the input microblog post set. It is shown as “Topic list” in Fig 1.

$$\begin{array}{c} E\left(A,B,D\right)=\{\left(d,s\right)|d\in D\wedge s\in R_{\geq 0}\wedge s=\Gamma \left(\beta \left(\alpha \left(A,B\right),d\right),\beta \left(d,\alpha \left(A,B\right)\right)\right)\} \end{array}$$ (2)

$$\begin{array}{c} \alpha (A,B)=\{(t,r)|\exists s,n[(t,s)\in A\wedge (t,n)\in B\wedge r=sn]\} \end{array}$$ (3)

$$\begin{array}{c} \beta (X,Y)=X\cup \{(t,0)|\exists p[(t,p)\in Y]\wedge \forall p[(t,p)\notin X]\} \end{array}$$ (4)

$$\begin{array}{c} \Gamma \left(X,Y\right)=\frac{\sum_{\left(t_{1},r\right)\in X,\left(t_{2},q\right)\in Y}\delta_{t_{1},t_{2}}rq}{\sqrt{\sum_{(t,p)\in X}p^{2}}\sqrt{\sum_{(t,p)\in Y}p^{2}}} \end{array}$$ (5)

$$\begin{array}{c} \delta_{a,b}=\{\begin{array}{cc} 1 & a=b\\ 0 & otherwise \end{array} \end{array}$$ (6)

Formulas (2)–(6) describe how the cosine similarity vectors are constructed. The Γ function (Formula 5) computes the cosine similarity given two sets’ tokens and *tf-idf* values. In our context, these sets correspond to a tweet set and a Wikipedia article. One of these sets is obtained from Twitter and the other from Wikipedia (a Wikipedia article). The *β* function (Formula 4) constructs the arguments for the Γ function. It adds the tokens that exist only in the first set to the second set and sets their *tf-idf* values in the second set to zero. The cosine similarity function takes into account both the presence and the absence of tokens. The presence of common terms contribute to increasing the similarity between the two sets, whereas the absence of a term in one of the sets results in decrease in cosine similarity, due to the normalization factor in the denominator of Formula 5. The *β* function is called twice with the *tf-idf* sets that represent the tweet set and the Wikipedia article by swapping their order between the calls.

To better explain, we give a simplified example. Let set *A* consist of only the words *church, catholic, abortion*, and *health*, and their corresponding *idf* values which is *A* = {(“*church*”, 0.23), (“*catholic*”, 0.27), (“*abortion*”, 0.475), (“*health*”, 0.53)}. Let set *B*, which is assumed to be extracted from the input microblog post set be *B* = {(“*church*”, 2), (“*catholic*”, 2), (“*abortion*”, 2), (“*health*”, 1)}. Let there be only two articles in Wikipedia which are “Christianity and abortion” and “Obamacare”. Let the sets representing these articles with the words and their corresponding *tf-idf* values be {(“*abortion*”, 0.76), (“*church*”, 0.68), (“*health*”, 0.23), (“*catholic*”, 0.55)} and {(“*health*”, 0.93), (“*obamacare*”, 1.0), (“*barack*”, 0.9)} respectively. Thus, set *D* is *D* = {{(“*abortion*”, 0.76), (“*church*”, 0.68), (“*health*”, 0.23), (“*catholic*”, 0.55)}, {(“*health*”, 0.93), (“*obamacare*”, 1.0), (“*barack*”, 0.9)}}

In this simplified environment, once the formulas are applied, *E*(*A*, *B*, *D*) is computed as

$$\begin{array}{ccc} E(A,B,D)=\{ & & \\ (\{(“health”,0.93),(“obamacare”,1.0),(“barack”,0.9)\},0.23), & & \\ \left(\{(“abortion”,0.76),(“church”,0.68),(“health”,0.23),(“catholic”,0.55)\},0.94\right)\\ \} \end{array}$$

The computation gives a relevancy score for the representative set of “Christianity and abortion” as 0.94 and that of “Obamacare” as 0.23. As observed from the set *B*, this is expected since its content is more similar to the representative set of the “Christianity and abortion” article.

Algorithm 1 shows the computation steps of the formal explanations.

**Algorithm 1** Topic identification algorithm

Input: A,B,D

Output: E // A set of Topics (Wikipedia articles) and their similarity scores.

**define** empty set F (each element is in (t,r) form)

**for each** (*t*_1_, n) in B **do**

 //in all input microblog post set tokens and their frequencies

 **for each** (*t*_2_, p) in A **do**

  //in all microblog idf set tokens and idf values

  **if** *t*_1_ = *t*_2_ **then**

  add (*t*_1_, n*p) to F // compute tf*idf value for the token and add to F

  **end if**

 **end for**

**end for**

**for each** d in D **do**

 //for each candidate topic (article)

 x,y,z ← 0

 **for each** (*t*_1_, p) in d **do**

  **for each** (*t*_2_, r) in F **do**

   **if** *t*_1_ = *t*_2_ **then**

   x ← x + p*r

   **end if**

  **end for**

  y ← y + p*p

 **end for**

 **for each** (*t*, r) in F **do**

  z ← z + r*r

 **end for**

 add (d,$\frac{x}{\sqrt{y}*\sqrt{z}}$) to E

 // compute article’s cosine similarity with F and add to E

**end for**

**return** E

## Implementation

We have implemented the approach similar to that in Algorithm 1. However, we have simplified the approach due to the large number of pages in Wikipedia. According to Algorithm 1, for all Wikipedia articles, the similarity operation should be applied. However, this is not feasible, since there are more than four million Wikipedia articles. For simplicity, we filtered out some of the articles. From the input post set words (set *B*), we have chosen top *μ* tokens according to their *tf-idf* values (*r* in *α*(*A*, *B*)). Pages which do not include any of these tokens are filtered out. To achieve this, we have first indexed all Wikipedia article contents in Solr (Solr is a document indexing program with a web service based on the Lucene index. See http://lucene.apache.org/solr/ for more details). Solr is then queried for the selected tokens. This way, Solr returns a subset of Wikipedia articles that are related to distinctive words (words with higher *tf-idf* scores) in the input microblog post set. The implemented algorithm computes the cosine similarities for only the Wikipedia pages returned by Solr.

It can be interpreted from the formal explanations in the approach section that, once a Wikipedia article is transformed in to tokens and their *tf-idf* value pair sets, the set loses reference to the original article. In our implementation, this is avoided. While operations are carried out, the algorithm keeps track of which *d* ∈ *D* corresponds to which specific Wikipedia article.

We used the Wikipedia dump which was taken on August 5, 2013 as the external source. We employed syn-sets in WordNet 3.1 [11] for enriching the Wikipedia topics. This is done to improve the chances of detecting a relationship between a topic and a microblog post set. To achieve this, words in the Wikipedia article titles are queried in WordNet. In order to select the relevant syn-set for a query term, each syn-set is compared against the words and their *tf-idf* values of the Wikipedia article using cosine similarity. All words in the selected syn-set are inserted to the corresponding set *d* of the article in *D*. The value for the words are set as the same as the query term. This assigns the words equal importance as the query term used to query WordNet.

We retrieved a dataset via Twitter API for computing *idf* scores of words in a microblog environment (“Twitter idf store” in Fig 1). The dataset consists of 7,347,669 microblog posts starting from July 11, 2013, 08:57am (GMT) to July 16, 2013, 03:30am (GMT). The microblog posts are retrieved by setting the Twitter API parameters as a sample of English microblog posts.

The most frequent word in the collected dataset is “rt” which is a common word that indicates a retweet. This word exists in 30 percent of the microblog posts. Some of the most common words like “the” (%24), “to” (%22), “and” (%14), and “not” (%4) are already in our stopwords list. Some of the other most common words were pronoun related terms like “you” (%22), “my” (%12), “me” (%10), “i’m” (%7), and “your” (%5).

We have preprocessed (*i.e*. tokenized the text and eliminated the stopwords), indexed, and stored all Wikipedia articles, making this data ready to be used by the implemented system (Set *D*). Indexing word frequencies of Wikipedia with PostgreSQL database management system took 41 gigabytes, indexing how many articles a word exists in, all articles in Wikipedia, took 408 megabytes, and indexing the number of microblog posts a word exists in, for all words in microblogs, took 199 megabytes of disk space (used to create the set *A*). The Solr index took 21 gigabytes of disk space.

The average length of a Wikipedia article is 590 words [38]. Thus, it is safe to assume that, the average number of unique tokens in a Wikipedia article is equal to or below 590. The average number of unique tokens in a two-minute debate in our dataset is 8200. Thus, the cosine similarity operation is applied on vector sizes of average 9000.

## Experiments and Results

We performed our experiments on datasets retrieved from Twitter, during the presidential debates and the vice presidential debate while the 2012 United States elections campaigns were run.

We refer to an interval of minutes using [t1,t2) to indicate a time interval beginning with t1 until (but not including) t2.

First we introduce the debates’ datasets that are used for evaluation and analysis. Next, we provide the experiments performed and the results obtained.

### Datasets

We retrieved the three presidential debates’ and the vice presidential debate’s dataset using Twitter’s streaming API, employing the streaming API’s filter endpoint [39]. The filter endpoint uses a set of keywords to filter tweets, returning tweets that include at least one of the keywords in the set. For the first, second and third presidential debates, we used the keywords {“obama”, “romney”, “barack”, “mitt”, “republican”, “democrat”, “elections2012”}. For the vice presidential debate, we added the keywords {“joe”, “paul”, “biden”, “ryan”, “vpdebate”} to the former. The datasets and their features are given in Table 1. For each debate microblog posts were continuously retrieved throughout the duration of the debate from the Twitter stream with a query. The queries for each debate are given in Section “Datasets”. Tweets associated with each debate were partitioned into two minute segments resulting in 45 segments per debate where each segments is identified as [0-2), [2,4),.. [88,90). In total, there are 45 × 4 = 180 segments.

**Table 1:** Features of the three Presidential Debates (PD) and the Vice PD datasets.

Debate	Start time (GMT)	End time (GMT)	# of tweets	# of users	# of tokens	# of unique tokens
1st PD	Oct 04, 2012 02:00:00	Oct 04, 2012 03:29:59	269,990	222,261	2,035,180	149,691
Vice PD	Oct 12, 2012 02:00:00	Oct 12, 2012 03:29:59	270,003	181,854	2,132,895	114,285
2nd PD	Oct 17, 2012 02:00:00	Oct 17, 2012 03:29:59	269,970	222,300	2,057,734	141,905
3rd PD	Oct 23, 2012 02:00:00	Oct 23, 2012 03:29:59	270,018	202,340	2,217,658	128,599

### Experiments

We identified the topics of microblog posts in two-minute intervals. We formed a microblog post set for each interval. Therefore, we obtained 45 sets for each debate. In total, we obtained 180 sets. The topics of a 90-minute debate (45 sets) could be identified in about 40 minutes by a Pentium-IV 3.2 GHz. computer with 4 GB of RAM.

**Fig 2:**
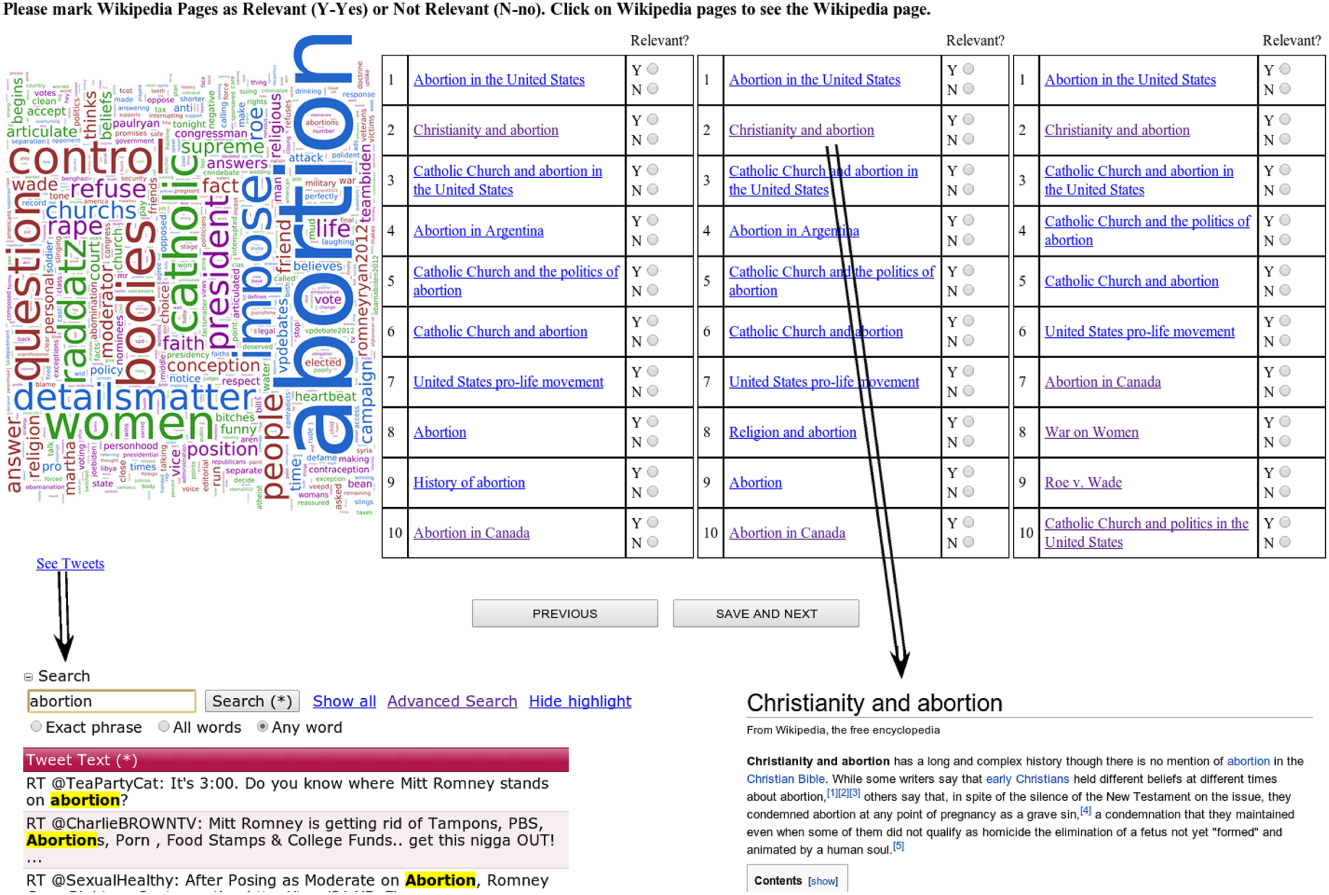
The evaluation interface. Evaluators used the interface at the top to annotate results as relevant or irrelevant to the input microblog post set. Since there are about 6000 microblog posts in each set, it is hard for an human evaluator to look at each microblog post. To give a general view of the microblog post set, we showed a word cloud to the evaluators. Evaluators used the “See Tweets” link to see tweets that led to these results. In the “See Tweets” interface a keyword based search tool is present and helped evaluators find tweets based on keywords of interest. The topics could be explored by clicking on the word and observing the contents of the corresponding Wikipedia topic.

We asked human evaluators to annotate the topics, i.e. the Wikipedia pages returned by the system, as relevant or not to the input microblog post set. To allow for annotation, we implemented an annotation interface. We created a representative word cloud for the microblog post sets, showing the three topic rankings to the evaluators. Lists were formed by taking the threshold *μ* as *μ* = 20, *μ* = 50 and *μ* = 100 in order to investigate the effect of the pre-filtering of topics using Solr. (See *Implementation* section for the description of the *μ* parameter). The top ten scored topics for each list were shown to the evaluators. A view of the evaluation interface is given in Fig 2.

In the experiments, we randomly selected 30 sets for evaluation. Initial analysis showed that most of the results contained “Barack Obama” and “Mitt Romney” Wikipedia page titles as top scored titles. This is not surprising since the datasets were retrieved using the words in the content of the pages. We removed these topics from the results. In addition, we removed the topics which have titles that included the keywords and their plural forms that we have queried Twitter API with. Finally, these words were also removed from the word clouds.

Two evaluators then annotated the results. We showed each evaluator twenty microblog post sets and their corresponding results. Ten of the microblog post sets were the same for both evaluators in order to calculate the inter-annotator agreement rate.

### Evaluation results

We randomly selected thirty tweet sets from the 180 tweet sets of the four debates. The distribution of these thirty tweet sets across time and debates is as follows: for the first presidential debate: [18,20), [26,28), [30,32), [40,42), [50,52), [54,56), and [84,86); for the second presidential debate: [26,28), [32,34), [44,46), [50,52), [56,58), [60,62), [74,76), and [88,90); for the third presidential debate: [18,20), [20,22), [26,28), [38,40), [40,42), [48,50), and [82,84); and for the vice presidential debate: [8,10), [16,18), [30,32), [36,38), [68,70), [80,82), [84,86), and [88,90). As our evaluation metric, we used precision scores obtained for the top *φ* topics returned by the proposed method, where *φ* was set as 1, 5, and 10. The precision for each tweet set was computed as the ratio of the number of true positives over *φ* (i.e. the number of all topics that were shown to the annotators). The results achieved are shown in Table 2. Finally, we give inter-annotator agreement *F*_1_ measures in Table 3. We calculated the *F*_1_ measures as given by Hripcsak and Rothschild [40].

**Table 2:** Precision scores of the top 1, 5, and 10 topics according to *μ* parameter.

	*μ* = 20	*μ* = 50	*μ* = 100
(*ϕ* = 1) top 1	0.90	0.90	0.96
(*ϕ* = 5) top 5	0.86	0.89	0.89
(*ϕ* = 10) top 10	0.82	0.80	0.83

**Table 3:** Inter-annotator agreement *F*_1_ measure of the top 1, 5, and 10 topics according to *μ* parameter.

	*μ* = 20	*μ* = 50	*μ* = 100
(*ϕ* = 1) top 1	1.00	1.00	1.00
(*ϕ* = 5) top 5	0.96	0.95	0.93
(*ϕ* = 10) top 10	0.93	0.91	0.89

The results indicate that, among the evaluated *μ* values, *μ* = 100 performs the best. The system achieved 0.96 precision. However, precision drops while *ϕ* increases. This shows that, top ranked Wikipedia pages are able to represent the tweet contents effectively. Inter-annotator agreement rates confirm this condition. They also drop while *ϕ* increases. More robust and better results are achieved if the top ranked topics are taken into consideration.

## Discussions

In this section, we investigate the debate datasets and the topics detected to better explain the impact of our approach.

**Fig 3:**
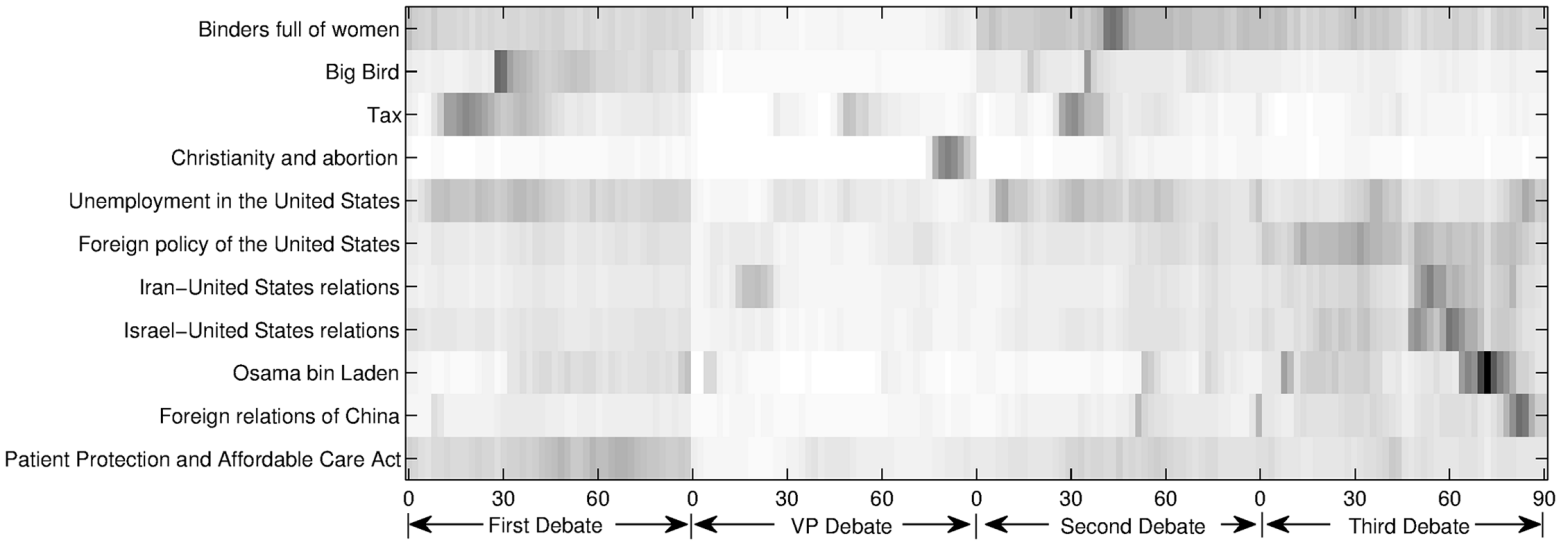
Heatmap of topics in the four debates. *x* axis shows the minutes of debates and *y* axis shows a selection of topics. These topics are among the top ranked topics when the approach is applied to the datasets. The darker the color, the more the topic is talked in the corresponding minutes.

In Fig 3, we give some topics and their scores in two-minute-interval microblog post sets on a heatmap. In order to obtain this heatmap, we have applied the cosine similarity function over the entirety of the debates, but for specific topics. We have selected some of these topics to show in the figure. We show a topic if it is either one of the highest ranked topics at some time in the debate, or it showed significant difference during different debates.

**Fig 4:**
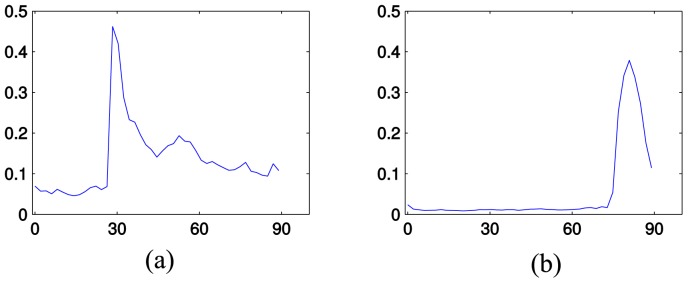
Two topics and their scores over time in two different debates. *y* axis is the score of the topic and *x* axis is the time in minutes. (a) “Big Bird” in the first debate. (b) “Christianity and abortion” in the vice presidential debate.

In Fig 3, we can observe that some topics suddenly appear, like “Big Bird” and “Christianity and abortion”. Mitt Romney mentioned “Big Bird” in the first presidential debate. He said that, if he is elected, he would cut the subsidy to the Public Broadcasting Service (PBS). He said that he loves “Big Bird” but he does not want to spend money on it. This speech received a quick response from microbloggers. For ease in observing the difference, Fig 4a focuses on this topic. The sudden change can be seen around the 28th minute of the debate. The transcription of the debate from the New York Times web site reports that Mitt Romney made these statements in the 26th minute (4–5 seconds after the 1564th second) of the debate. According to CNN web site, the debate started 100 seconds after Oct 04, 2012 02:00:00 (GMT). When this is considered, we can conclude that just a few seconds after Mitt Romney made this statement, the effect is immediately seen in the microblogging environment. The same topic is observed in the second debate around 15th and 40th minutes. This is unexpected once we observe the debate transcripts. This shows that people may continue to talk about other topics even though the opponents have moved on and talk about other issues.

“Binders full of Women” is a phrase Mitt Romney used in the second presidential debate. It received intense attention from microbloggers. Its effect continued in the third presidential debate as shown in Fig 3.

The scores of the topics “Unemployment in the United States” and “Tax” showed similar behavior except in the beginning part of the second debate. The reason may be, according to the transcripts, that during the beginning, the opponents talked about unemployment issues but not explicitly about tax issues.

“Patient Protection and Affordable Care Act”, also known as “Obamacare”, received higher scores in the first debate since it was mostly in the first debate, that opponents discussed health care related issues and Obamacare specifically.

The third debate focused on foreign relationships and issues. The “Foreign policy of the United States” topic, received higher scores than it had in the other debates. This shows that, in practice, the approach gives reasonable results. For instance, we can summarize the second half of this debate by looking at the heatmap. The opponents talked about these topics in order: “Israel-United States relations”, “Iran-United States relations”, “Israel-United States relations” (again), “Osama bin Laden”, and “Foreign relations of China”. All these topics scored the highest in the minutes when the opponents were talking about them. In the minutes “Iran-United States relations” is ranked the first, the second topic was “Views on the nuclear program of Iran” which was also one of the related topics the opponents discussed. It is further observed that when opponents talked about “Iran-United States relations” in the vice presidential debate the results are similar in the analysis and is confirmed by looking at the debate transcriptions.

In the vice presidential debate, the moderator asked the opponents about their position on abortion as Catholics. This topic quickly received attention from microbloggers as observed after the 74th minute in Fig 4b. The “Christianity and abortion” topic scored at the top of the analysis in the 80th minute. Other topics ranked below this topic in order were “Abortion in the United States”, “Catholic Church and abortion in the United States”, and “Catholic Church and abortion”. These were topics were all related to the main discussion.

It is important to mention that, “Christianity and abortion”, and “Reactions to the death of Osama Bin Laden” were not phrases used in microblog posts specifically. The approach reveals these topics are drawn out of the Wikipedia article titles. Studies that only consider titles of the Wikipedia articles cannot reveal these topics, but would reveal Osama Bin Laden, Catholic Church, and Abortion separately, as the dataset confirms that these words were frequent.

Often there are dominating topics in the two minutes segments, where the identified topics are very similar. However, in segments where the contributions center among multiple subjects, varying topics are identified. For example, in the [62,64) minutes of the third presidential debate, the highest ranking topic is “Israel–United States relations”. The second and third ranking topics are “Reactions to the death of Osama bin Laden” and “Osama bin Laden”. In the [46,48) minutes of the first presidential debate the top ranking topics are “Medicare (United States)”, “United States presidential election”, and “The Path to Prosperity”.

In this paper our goal was to identify the main topics in a microblog post set represented with Wikipedia page titles. We did not explicitly tackle the problem of identifying sub-topics, which may be represented with subsections in a Wikipedia page. It is interesting to observe that, sometimes topics that appear as subsections of a Wikipedia article, have a corresponding independent page. For such cases, if the microblog posts set content is more focused on the sub-topic, cosine similarity will result in a higher similarity score for the independent page and our approach will be able to identify the corresponding sub-topic.

Considering the evaluation results, and observations in this section, we can conclude that the proposed approach achieves promising results and is suitable for further research and study.

### Comparison of processing single-microblog posts and microblog post sets

In this section, we compare processing single-microblog posts to our approach.

One of the state of the art approaches in the analysis of single microblog posts is put forward by Ferragina and Scaiella [34, 35, 41] (Tag.Me). This approach attempts to annotate parts of a microblog text with a Wikipedia page. Wikipedia titles are utilized to identify candidate topics from text fragments. Candidate Wikipedia pages for the text fragment are identified based on their popularity. One of the pages is picked according to the context of the fragment. In order to investigate what such an approach would yield for our post sets, we considered aggregating the result from the elements of this set by selecting the Wikipedia articles with the highest frequency. Then, we compared these results with the topics identified by our approach BOUN-TI.

We applied two different microblog post sets in our datasets to compare the results. Tables 4 and 5 show the top five topics obtained by the two systems. The results were obtained by applying the microblog posts in [28,30) minutes of the first presidential debate and [80,82) minutes of the vice presidential debate, respectively.

**Table 4:** Comparison of topics identified by the aggregation of Tag.Me topics and BOUN-TI for the [28,30) interval of the first presidential debate.

Rank	Tag.Me	BOUN-TI
1	Big Bird	Big Bird
2	Lava	Bush tax cuts
3	(F word)	Economic policy of the George W. Bush administration
4	PBS	Tax Relief, Unemployment Insurance Reauthorization, and Job Creation Act of 2010
5	You (Time Person of the Year)	United States presidential election, 2012

**Table 5:** Comparison of topics identified by the aggregation of Tag.Me topics and BOUN-TI for the [80,82) interval of the vice presidential debate.

Rank	Tag.Me	BOUN-TI
1	Abortion	Christianity and abortion
2	Catholic Church	Abortion in the United States
3	Belief	Catholic Church and abortion in the United States
4	Transmitter	Catholic Church and abortion
5	People	Abortion in Argentina

For the [28,30) minutes of the first debate, both approaches returned “Big Bird” as the first ranked topic. Tag.Me returned this output since the words “Big” and “Bird” appeared in the same microblog post consecutively many times. Applying the approach to each microblog post returned this Wikipedia page the most number of times so it was ranked first. But, our approach, BOUN-TI, returned “Big Bird” as the first ranked since words “Big” and “Bird” appeared many times in the microblog post set regardless of sequentiality in the same microblog post. “PBS”, which was the fourth ranked topic in the results of the single microblog post processing approach, was captured by this approach. People mainly mentioned “PBS” in the context of the “Big Bird” topic. Other topics in the list of Tag.Me are not related to the topics talked about in those times during the first debate. All topics returned by our proposed microblog post set processing approach were related to the input microblog post set. This is due to the similarity of the words in the microblog post set and Wikipedia pages.

For the [80,82) minutes of the vice presidential debate, both approaches returned relevant results. Tag.Me returned separate pages as results such as “Abortion” and “Catholic Church”, while BOUN-TI returned inclusive and descriptive results such as “Christianity and abortion” and “Abortion in the United States”. This is due to the nature of our approach since it considers all the words in the microblog post set unlike Tag.Me which considers only words that are in the same microblog post. The topic “Abortion in Argentina” is not relevant but appears in the top five results of our approach. This is a false positive that resulted due to the frequent occurrence of the term “abortion” in the corresponding Wikipedia page. At capturing the topics, these results suggest that post set processing is in general better than processing single posts and aggregating.

## Future Work and Conclusions

First, we explain possible future work on this approach and then conclude the study. Error analysis reveals that the false positive topics are pages that are related to the topic in general, however in a different context. For instance, in the abortion example, the “Abortion in Argentina” and “Abortion in Canada” topics were in the top 10. These topics received high scores since the word “abortion” is frequently used on Twitter. These Wikipedia articles also have “abortion” as one of the highest *tf-idf* values. This is the main reason they received high scores. This issue, false positive topics that rank highly, should be addressed in future work. This issue can be solved by adding co-occurrence sensitivity to the computation. For instance, ideas similar to [32], which considers the consecutiveness of word pairs, or [16], which considers co-occurrence of word pairs in the same post can aslo be considered. Word pairs can be added to the representative sets to further filter the data more effectively. Wikipedia pages include headings and subheadings. Another future work is investigating whether the headings and sub headings can be an identifier for a topic given a microblog post set.

Approaches for identifying finer grained subjects based on subsections of articles should be investigated. In such cases the scope of the match within the Wikipedia article should be explored.

To show the strength of the approach, it can be applied to further datasets.

Social network analysis can be applied, to better identify sources of information. For instance, microblog posts posted by socially close users may give better results in identification.

To conclude, in this study, we introduced an approach to automatically identify topics in a collection of microblog posts. Existing related works that dealt with topic identification in microblogs either only considered a single microblog post, or returned outputs that required further manual analysis or interpretation. With an experiment provide an example of our approach, we show that, considering only one microblog post may miss the overall context of a topic. This makes our approach stronger since it derives topics from a whole microblog post set. Our approach also differs from existing approaches by its type of output which is a human readable user generated Wikipedia page title. Although, our approach is bounded within the coverage of topics contained in Wikipedia, its breadth is relatively high with over four million Wikipedia pages if topics of related works are also considered.

We applied the proposed approach and gave an evaluation of the results. We evaluated the system of microblog post sets retrieved by a number of keywords while the 2012 U.S. presidential and the vice presidential debate were happening. Results were evaluated by human annotators. Topics identified at the top received a 0.96 precision with inter-annotator agreement *F*_1_ measure *F*_1_ = 1. This shows that our approach achieved promising results in identifying relevant Wikipedia page titles against a given set of microblog posts.

Evaluation is a challenge in this domain, since it is difficult to manually annotate huge numbers of microblog posts. We obtained a manually annotated dataset that could be used for further research and evaluation. Finally, we have also qualitatively shown in the discussion section that, our approach identifies topics effectively.
